# Can sodium-glucose cotransporter-2 inhibitors stabilize coronary atherosclerotic plaques? Evidence from imaging studies

**DOI:** 10.1093/ehjcvp/pvaf025

**Published:** 2025-04-12

**Authors:** Filippo Luca Gurgoglione, Filippo Crea, Giampaolo Niccoli, Mattia Galli

**Affiliations:** Division of Cardiology, University of Parma, Parma University Hospital, 43100 Parma, Italy; Center of Excellence of Cardiovascular Sciences, Ospedale Isola Tiberina—Gemelli Isola, 00100 Rome, Italy; Catholic University of the Sacred Heart, 00168 Rome, Italy; Division of Cardiology, University of Parma, Parma University Hospital, 43100 Parma, Italy; Department of Medical-Surgical Sciences and Biotechnologies, Sapienza University of Rome, Corso della Repubblica, 79, 04100 Latina, Italy; Maria Cecilia Hospital, GVM Care & Research, 48033 Cotignola, Italy

Sodium-glucose cotransporter-2 inhibitors (SGLT2i) have become a cornerstone in the management of Type 2 diabetes mellitus (T2DM) and heart failure (HF). Among patients with chronic coronary syndrome (CCS), European Society of Cardiology guidelines recommend SGLT2i in patients with T2DM due to their cardiovascular (CV) benefits.^1^ While the mechanisms underlying the benefits of SGLT2i in patients with T2DM and HF are better understood, their cardioprotective effects in patients with CCS remain less clearly defined. Emerging evidence suggests that SGLT2i may favourably modulate coronary plaque composition and stability, potentially contributing to improved CV outcomes in this population.

Sardu *et al*.^2^ evaluated 369 T2DM patients with CCS and multivessel non-obstructive coronary artery disease (CAD) using optical coherence tomography (OCT). Sodium-glucose cotransporter-2 inhibitor users exhibited significantly higher minimum fibrous cap (FC) thickness, reduced lipid arc, and lower macrophage grade compared with non-SGLT2i users at the 1-year OCT follow-up. Notably, results were consistent across different SGLT2i, suggesting a potential class effect.^2^ A small OCT study further supported the plaque-stabilizing effects of SGLT2i in patients with acute coronary syndrome.^3^ In this study, SGLT2i users exhibited thicker FC and reduced lipid arc in non-culprit lesions after 6 months of treatment, compared with non-SGLT2i users.^3^ Additionally, a longitudinal computed tomography angiography study showed that SGLT2i reduced coronary plaque volume in 236 T2DM patients with non-obstructive CAD at baseline treated with SGLT2i for a mean of 14.6 months.^4^

The observational design of these studies limits interpretation, underscoring the need for randomized trials to determine whether SGLT2i may stabilize or reverse coronary plaques. If confirmed, such effects could offer insights into the mechanisms underlying the cardioprotective effects of SGLT2i in patients with CAD, potentially supporting their broader use in both diabetic and non-diabetic populations.

## Data Availability

There are no new data associated with this article.
